# Efficacy of thoracotomy and thoracoscopic-assisted esophageal surgery in conversion and salvage surgeries: a retrospective study

**DOI:** 10.1186/s12957-022-02637-8

**Published:** 2022-05-23

**Authors:** Isamu Hoshino, Hisashi Gunji, Naoki Kuwayama, Takeshi Kurosaki, Toru Tonooka, Hiroaki Soda, Nobuhiro Takiguchi, Yoshihiro Nabeya, Wataru Takayama

**Affiliations:** Division of Gastrointestinal Surgery, Chiba Cancer Center, 666-2 Nitona-cho, Chuo-ku, Chiba, 260-8717 Japan

**Keywords:** Esophageal cancer, Conversion surgery, Salvage therapy, Chemotherapy, Chemoradiotherapy

## Abstract

**Background:**

The esophagus has no serosa; therefore, esophageal cancer may quickly invade its adjacent organs. In recent years, reports of conversion surgery (CS) and salvage surgery (SS) have described resection of esophageal cancer previously considered unresectable, with the addition of intensive preoperative chemotherapy or chemoradiotherapy. Currently, there is no established method for determining whether tumor excision is possible. Additionally, differences in surgical approaches between facilities may influence outcome after resection. However, the option for resection is considered a significant factor in determining a patient’s prognosis.

**Methods:**

Patients who were diagnosed with advanced-stage (T3 or higher) squamous cell carcinoma of the esophagus and subsequently underwent resection with CS or SS were included in the study. Resection was performed through a small thoracotomy using a thoracoscope. Clinicopathologic factors, such as complete resection rate (R0) and prognosis, were investigated.

**Results:**

A total of 49 surgeries were conducted: 39 CS and 10 SS cases. The male-to-female ratio was 37:12. R0:R1:R2 equals 42:3:4, and the R0 resection rate was 85.7%. The 5-year survival rates for CS and SS cases were 69.2% and 32.1%, respectively. The 5-year survival rates for R0, R1, and R2 resections were 63.4%, 0.0%, and 25.0%, and those for R0 and R1 + 2 resections were 63.4% and 14.3%, respectively, indicating that the prognosis for R0 resection cases was significantly better (*P* = 0.001 and *P* = 0.001, respectively). Regarding chemotherapy for CS, 29 patients received 5-FU and cisplatin therapy, whereas 10 patients received 5-FU, cisplatin, and docetaxel (DCF) therapy. After 2015, the ratio of DCF was significantly high, and the R0 resection rate was 100% in patients who received DCF therapy.

**Conclusions:**

In this study, a satisfactory R0 rate was achieved using the magnifying effect of the thoracoscope while ensuring safety during thoracotomy.

**Trial registration:**

This was a single-center cohort study wherein clinical data were retrospectively registered. This study was approved by the Chiba Cancer Center review board (H29-262). All procedures adhered to the ethical standards of the responsible committee on human experimentation and the Helsinki Declaration of 1964 and its later amendments.

**Supplementary Information:**

The online version contains supplementary material available at 10.1186/s12957-022-02637-8.

## Background

Most of the esophagus is located vertically, adjacent to the trachea, bronchi, aorta, and heart, all of which are vital organs for life support [1]. In addition, there is no serosa in the esophageal wall, and it is thought that esophageal cancer invades adjacent organs as it progresses [2]. Diagnostic techniques have improved in recent years, and early diagnosis of esophageal cancer has become increasingly possible through examinations [3]. However, esophageal cancer is usually asymptomatic in the early stage and only detected in the advanced stage [4]. Consequently, it is often difficult to perform definitive resection in several of these cases. In recent years, however, conversion surgery (CS) has been increasingly performed, which allows resection of esophageal cancer previously considered unresectable with the addition of intensive preoperative chemotherapy. The use of salvage surgery (SS), which targets relapsed lesions or cancer remnants after radical chemoradiotherapy, has also increased [5–12]. Nonetheless, a diagnosis of T4 tumors is challenging, and even once a diagnosis has been made, it does not necessarily provide certainty [13]. Moreover, because of the differences in surgical approaches between institutions regarding CS and SS cases, no method has reached a clear consensus in the surgical community. In addition, a significant difference in prognosis is observed depending on whether radical resection can be achieved (Comprehensive registry in Japan, 2012, 5-year survival rate, complete resection rate [R0]:R1:R2 = 59.8%:19.4%:7.0%) [14]. The most important factor that determines a patient’s prognosis is the ability to receive radical resection without any residue [14]. However, especially in SS, the mortality rate is high; therefore, the procedure should be safely performed to prevent complications [15–21].

In recent years, thoracoscopic esophagectomy without thoracotomy and, more recently, robot-assisted esophagectomy have been gradually generalized, and there are reports of CS and SS using the thoracoscopic approach in particular [22].

In this study, we used a thoracoscopic approach with a small thoracotomy. We believe that this approach enables a prompt response to potential bleeding and organ damage. Another merit of this method is the magnifying effect of the thoracoscope. Here, we report the results of thoracoscopic esophagectomy under a small open-chest condition for CS and SS cases that we have performed so far.

## Methods

### Patients

This single-center, retrospective cohort study included 49 patients with clinically borderline unresectable T3, T4a, and T4b primary esophageal cancers without distant metastases who were referred to the Chiba Cancer Center between January 2007 and December 2019. Lymph node metastases are considered to indicate local lymph nodes in the Japanese esophageal cancer classification and were eligible for inclusion in this study. Patients with resectable tumors underwent radical esophagectomy with two or three fields of lymph node dissection. Preoperative induction chemotherapy included 5-FU and cisplatin (FP) therapy (70 mg/m^2^ of cisplatin on day 1 and 700 mg/m^2^ of 5-FU for two cycles on days 1–4) or 5-FU, cisplatin, and docetaxel (DCF) therapy (70 mg/m^2^ of cisplatin on day 1 and 700 mg/m^2^ of 5-FU for two cycles on days 1–4). In addition, for radical radiation therapy, chemoradiation therapy (CRT) with a total dose of 50–60 Gy was administered. After total irradiation, patients without progressive disease received two cycles of additional FP chemotherapy. If the tumor remained after the intervention, esophagectomy was performed. Patients with resectable tumors or those who had a complete response but then relapsed after follow-up also underwent esophagectomy. Tumor resection was reassessed on the basis of computed tomography (CT) findings, and esophagectomy was performed on the resectable tumor. However, patients with unresectable tumors, those with inadequate surgical tolerance, and those who preferred esophageal preservation did not undergo surgery. Hospital records were used retrospectively.

Data on patient clinical features, such as age, sex, oncological findings, and long-term outcomes, including prognosis, were obtained. Overall survival (OS) was defined as the time interval between treatment initiation and death, regardless of cause, and disease-free survival (DFS) was defined as the shortest time interval between treatment initiation and progressive disease, disease recurrence, or death. Clinical cancer stage was determined according to the International Union Against Cancer, 7th Edition, staging [15]. Responses to treatment were assessed according to the Response Evaluation Criteria in Solid Tumors guidelines [16]. Imaging evaluations and treatment decisions were confirmed by a medical panel of specialist surgeons, oncologists, and radiologists. The status of residual tumors was classified as follows: pR0, no residual tumor; pR1, microscopic residual tumor; and pR2, residual tumor visible to the naked eye. This study was conducted with the approval of the Ethics Committee of the Chiba Cancer Center in accordance with the principles of the Helsinki Declaration of 1964 and its later amendments.

### Diagnosis of clinical T4 and borderline unresectable T3 tumors

The diagnostic criteria for T4 tumors were protrusion of the tumor into the tracheal or bronchial lumen and obstruction of the fat surface in the triangular space between the aorta, esophagus, and spine on CT [17]. In addition, tumors with more than 90° of direct contact with the aorta and tumors with overt infiltration of adjacent organs were diagnosed as stage T4 [18]. Borderline, unresectable T3 tumors were defined as locally advanced esophageal cancer with suspected infiltration into adjacent organs that could not be clearly diagnosed as T4 disease [19]. Imaging information was obtained using upper gastrointestinal endoscopy, contrast-enhanced CT, and positron emission tomography. All diagnoses were confirmed by agreement on the medical panel between specialist surgeons, oncologists, and radiologists prior to the start of treatment.

### Surgical procedure and evaluation of complications

**Fig. 1:**
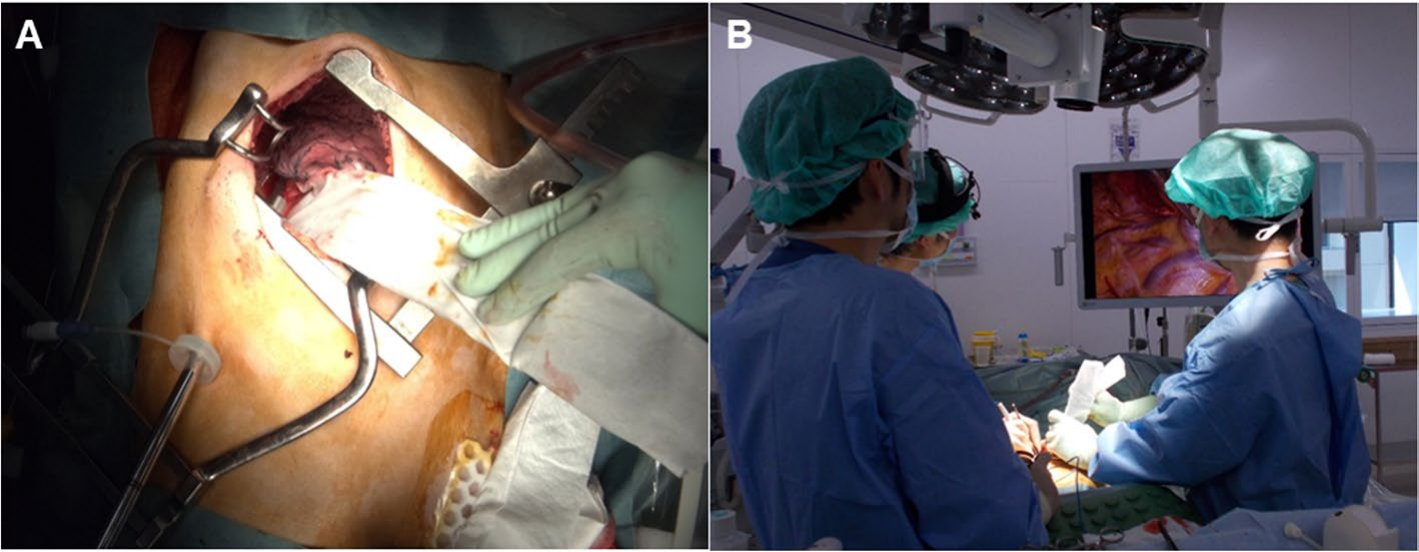
Thoracotomy with thoracoscopic assistance. **A** A 12-mm port was placed between the seventh intercostal line and the posterior axillary line, a flexible endoscope was inserted from the same site, and surgery was performed with thoracoscopic assistance. **B** The surgeon performs the operation while using the image on the monitor

All patients who underwent surgery underwent gastric tube reconstruction. Subtotal esophagectomy with local lymph node dissection by right thoracotomy with a thoracoscope and laparotomy, reconstruction by the posterior sternal route with cervical anastomosis by cervical incision, or reconstruction with intrathoracic anastomosis was performed (Fig. 1). In all cases, a 12-mm port was placed between the seventh intercostal line and the posterior axillary line, a flexible endoscope was inserted from the same site, and surgery was performed with thoracoscopic assistance.

The Clavien–Dindo classification was used to assess perioperative complications [22]. Simply put, grade II indicated the need for pharmacological treatment. Grade III indicated the need for surgical, endoscopic, or radiological intervention. Grade IV showed the presence of life-threatening complications requiring intensive care unit management. Grade V indicated death. Patients with major complications were defined as those with Clavien–Dindo grade III or higher complications. Anastomotic leakage was diagnosed on the basis of CT images or esophagography and/or the characteristics of the anastomotic drain. Hospital deaths were defined as deaths that occurred during hospitalization.

### Statistical analysis

All statistical calculations were performed using JMP Pro 13 software (SAS Institute, Cary, NC, USA). The OS and DFS rates were calculated from the date of surgery to the date of death due to any cause and first recurrence or death due to any cause, whichever occurred earlier. The survival curves were calculated using the Kaplan–Meier method. *P* values of <0.05 were considered statistically significant in all analyses.

## Results

### Patient characteristics and treatment overview

Table 1 presents the patient’s background. The mean age was 68 years, and there were 37 men and 12 women. The tumor location sites were Lt in 25 cases (51%) and Mt in 21 cases (42.9%). Regarding the preoperative tumor invasion depth, borderline T4 was classified as T3, and T3 was the highest in 40 cases (81.6%). Thirty-one cases (63.3%) had lymph node metastasis, accounting for the majority of cases. Ten patients (20.4%) received chemoradiotherapy, whereas 39 patients (79.6%) received preoperative chemotherapy. As a result, there were 39 CS cases (79.6%) and 10 SS cases (20.4%). The mean surgical time and amount of bleeding were 408 min and 336 mL, respectively. The mean length of hospital stay after surgery was 23 days. The most common complications of Clavien–Dindo II and above were pneumonia and recurrent laryngeal nerve palsy, both of which occurred in nine cases (18.4%). The postoperative in-hospital mortality rate was 0%.

**Table 1:** Patient characteristics and surgical outcomes

Demographics	Value
Age	68 (47–78)
Gender (male/female)	37 (75.5)/12 (24.5)
Tumor location (U/M/L)	3 (6.1)/21 (42.9)/25 (51.0)
Initial depth of tumor invasion (T1 or T2/T3/T4)	5 (10.2)/44 (89.8)
Initial nodal status (positive/negative)	18 (36.7)/31 (63.3)
Initial TNM stage (I/II/III/IV)	1 (2.0)/15 (30.6)/29 (59.2)/4 (8.2)
Treatment (CRT/CT)	10 (20.4)/39 (79.6)
CS/SS	39 (79.6)/10 (20.4)
Pathological depth of tumor invasion (T0–T2/T3/T4)	9 (18.4)/6 (12.2)/ 31 (63.3)/3 (6.1)
Pathological nodal status (positive/negative)	29 (59.2)/ 20 (40.8)
Resection margin (R0/R1/R2)	42 (85.7)/3 (6.1)/4 (8.2)
Operation time (min)	408 (250–732)
Bleeding (mL)	336 (40–2390)
Postoperative hospital stay (days)	23 (11–74)
Major postoperative complications (CDII<)	
Pneumonia	9 (18.4)
Anastomotic leak	2 (4.0)
Recurrent laryngeal nerve palsy	9 (18.4)
Surgery-related mortality	0 (0.0)

### Survival

**Fig. 2:**
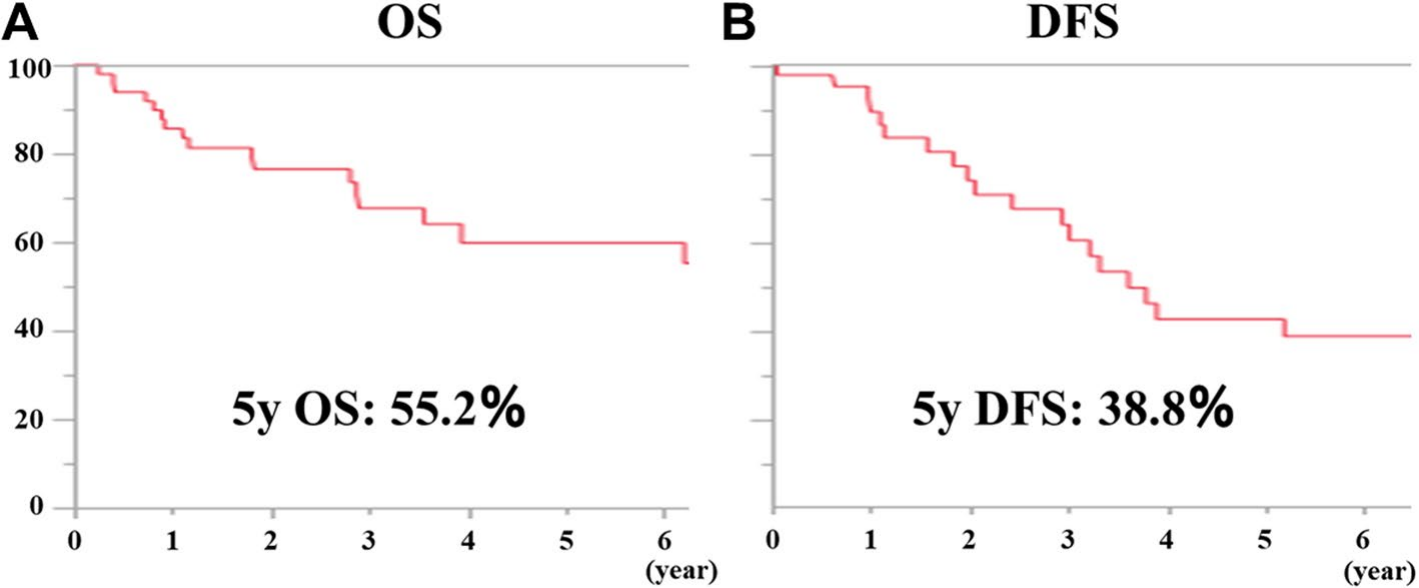
**A** The 5-year OS rate in all cases was 55.2%. B. The 5-year DFS rate in all cases was 38.8%

**Fig. 3:**
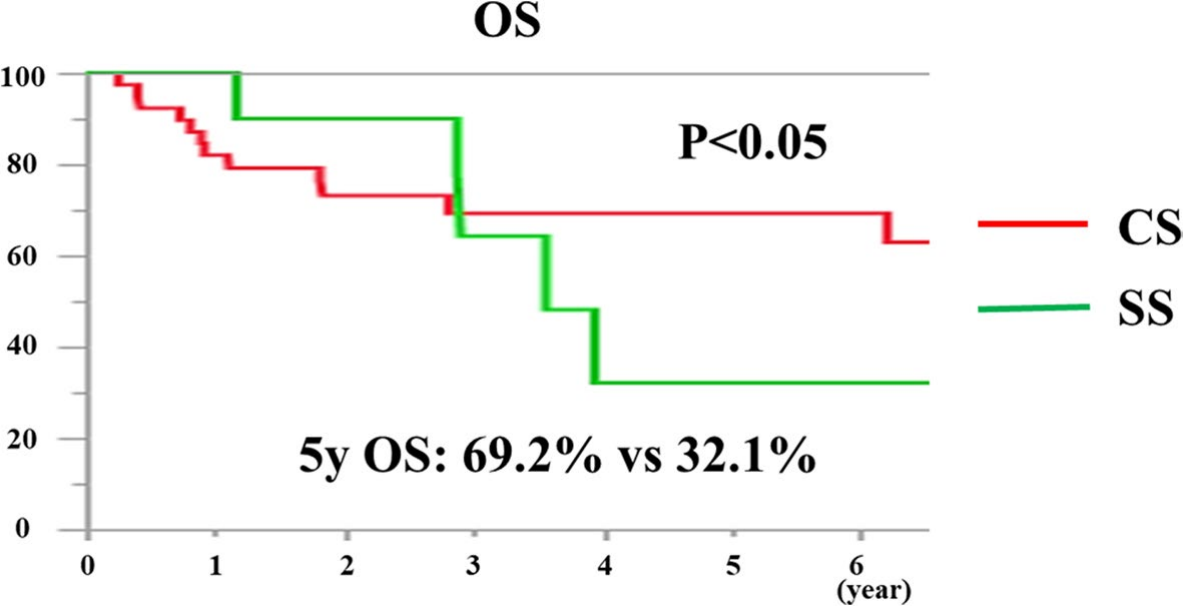
Comparing the survival rates of CS and SS cases separately, the 5-year OS rates were 69.2% and 32.1%, respectively (*P* < 0.05)

**Fig. 4:**
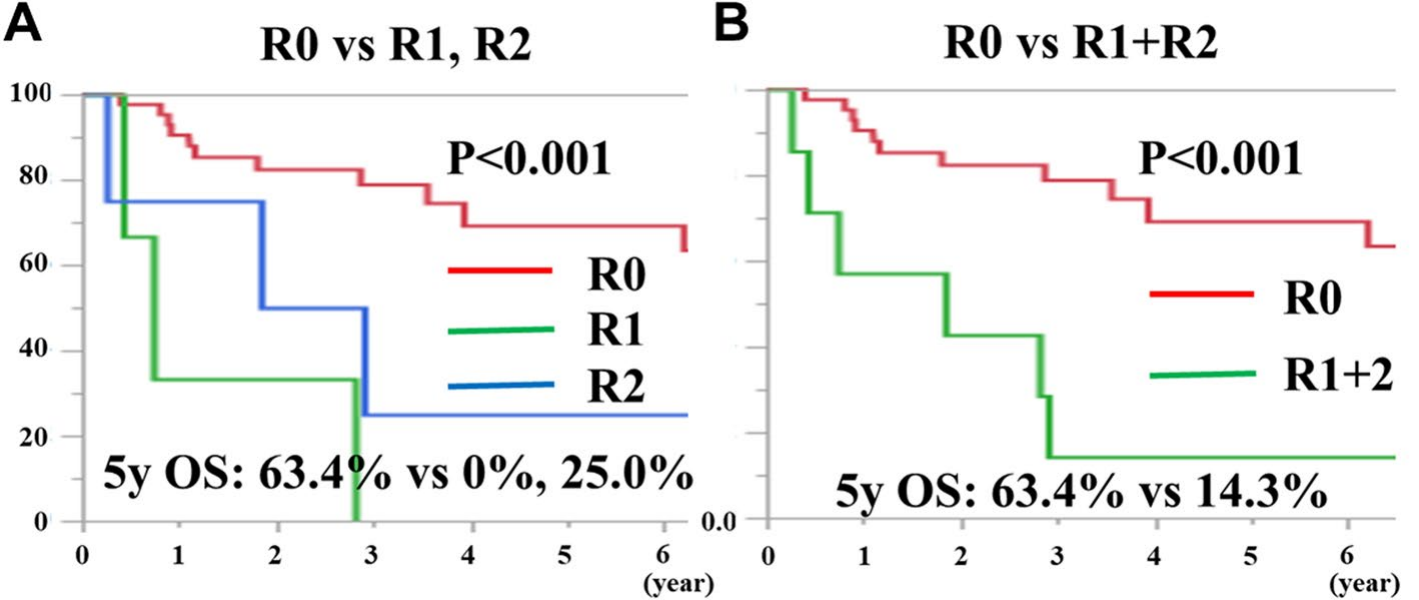
**A** The 5-year OS rates were 63.4%, 0.0%, and 25.0% for R0, R1, and R2, respectively, indicating that R0 excision was significantly better (*P* < 0.001). **B** In a separate study of R0 and R1 + R2, the 5-year OS rates were 63.4% and 14.3%, respectively, and the prognosis of R0 resection cases was significantly better (*P* < 0.001)

The median follow-up was 45.4 (range, 2.0–147.0) months for all patients. The 5-year OS and DFS rates in all cases were 55.2% and 38.8%, respectively (Fig. 2a and b). Comparing the survival rates of CS and SS cases separately, the 5-year OS rates were 69.2% and 32.1%, respectively, indicating that CS cases had a significantly better prognosis than SS cases (Fig. 3; *P* < 0.05). In addition, for the results of the section margin, the 5-year OS rates were 63.4%, 0.0%, and 25.0% for R0, R1, and R2, respectively, indicating that R0 excision was significantly better (Fig. 4a, *P* < 0.001). In a separate study of R0 and R1 + R2, the 5-year OS rates were 63.4% and 14.3%, respectively, and the prognosis of R0 resection cases was also significantly better (Fig. 4b, *P* < 0.001).

### Transition of preoperative treatment in CS cases

**Fig. 5:**
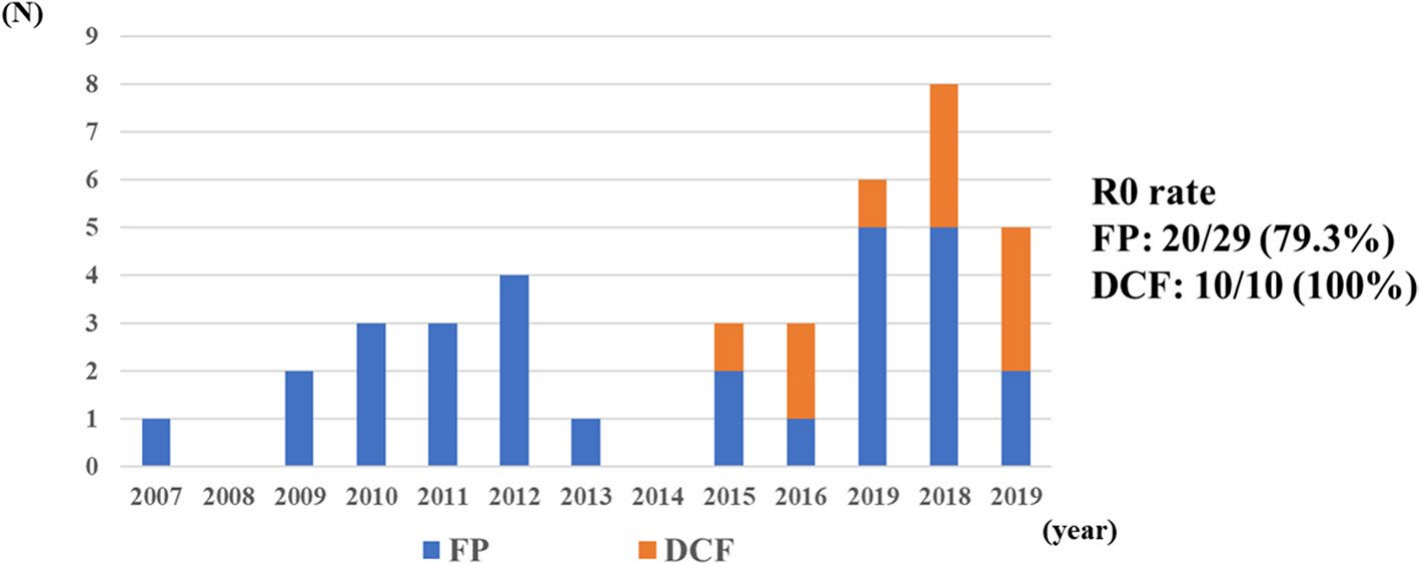
The transition of the preoperative chemotherapy regimen in patients with CS. Doublet chemotherapy (FP) has been the standard therapy, but in recent years, triplet chemotherapy (DCF) has become mainstream. In addition, R0 resection was performed in 23 (79.3%) of 29 patients who received FP therapy and in 10 (100%) of 10 patients who received DCF therapy

Figure 5 shows the transition of the preoperative chemotherapy regimen in patients with CS. Doublet chemotherapy (FP) has been the standard therapy, but in recent years, triplet chemotherapy (DCF) has become mainstream. In addition, R0 resection was performed in 23 (79.3%) of 29 patients who received FP therapy and in 10 of 10 patients who received DCF therapy.

## Discussion

This retrospective study examined 39 CS and 10 SS cases at our hospital. R0 resection was performed in 42 (85.7%) of 49 cases, which indicated a high R0 resection rate. The surgery-related mortality rate was 0%. In addition, although the number of CS patients who received DCF therapy has increased in recent years, here, R0 resection was performed all in patients who also received DCF therapy. Finally, the prognosis of R0 resection cases was significantly better than those of R1 and R2 resection cases.

Table 2 summarizes the reports of SS cases from other institutions thus far. The mortality rate was 7.4–15.2%, which was relatively high and varied between institutions. The mean mortality rate was 9.7%, which was higher than that of a general esophageal resection [23]. Meanwhile, at our institution, there were 10 SS cases, and the population was small; however, the mortality rate was 0%, indicating that the procedure was performed relatively safely. In addition, R0 resection was performed in 9 (90%) of 10 patients, and the resection rate was good. In addition, regarding the prognosis of SS cases, the 5-year OS rate was 5.7–50.6%, according to reports from various families, similar to the 32.1% noted in our institution. Although a large variation in the rates exists due to differences in patient background, these prognosis rates are relatively good. These results suggest that although SS cases require high-risk surgery with a potentially high mortality rate, the intervention may significantly help the prognosis, especially in cases where R0 resection is performed. For the surgery to be beneficial, it is important to emphasize safety and reduce complications. In that respect, a small thoracotomy using a thoracoscope is also useful from the viewpoint of the R0 resection achievement rate. In general, surgeries for SS cases are often performed under an open-chest condition. The use of a thoracoscope during the small thoracotomy enables the surgeon to perform delicate surgery by magnifying the fine anatomical structures. By proactively performing thoracotomy in advance, it is possible to immediately respond to an emergency and, at the same time, take advantage of the tactile sensation.

**Table 2:** Reports of SS cases from other institutions

	Author	Year	*N*	Mortality rate	5-year OS	Ref.
1	Nishimura [15]	2007	46	15.2	N/A	*Gen Thorac Cardiovasc Surg*
2	Tachimori [16]	2009	59	8.5	37.8	*J Thorac Cardiovasc Surg*
3	Miyata [17]	2009	33	12.1	35.0	*J Surg Oncol*
4	Takeuchi [18]	2010	25	8.0	43.0	*World J Surg*
5	Morita [19]	2011	27	7.4	50.6	*J Gastroenterol*
6	Watanabe [20]	2015	63	7.9	15.0	*Ann Surg Oncol*
7	Okamura [21]	2019	35	8.6	5.7	*Esophagus*

Conversely, for CS, although the background may be different, R0 resection was performed in 33 of 39 cases at our institution, and the R0 resection rate was 84.6%. As for the SS cases, the mortality rate was 0%, and the surgery was performed with presumed guaranteed safety. In recent years, there have been increasing reports on the usefulness of DCF therapy in patients with CS. Miyata et al. conducted a randomized controlled trial study of induction CRT and induction chemotherapy in patients with unresectable cT4b thoracic esophageal cancer [11]. For the induction chemotherapy cases, triplet DCF therapy was performed, as in our case. Induction DCF therapy was performed in 50 patients, and consequently, grade 3–4 leukopenia and neutropenia were observed in as frequently as 88% and 94%, respectively, and febrile neutropenia was also observed in as frequently as 52%. Furthermore, fistula formation was observed during the chemotherapy trials in two cases. However, a clinical response was observed in 26 of 50 cases (52%), and it was determined that resection was possible on the image in 42 of 50 cases (84%). Resection was performed in 41 cases, and R0 resection was achieved in 38 patients (93%). The postoperative complication rates of anastomotic insufficiency, pneumonia, and recurrent laryngeal nerve palsy were 11.1%, 27%, and 15%, respectively, which were considered acceptable results compared with the standard frequency of complications following general esophagectomy. They also performed esophagectomy with thoracotomy in 39 (95%) patients who underwent resection. Yokota et al. additionally reported the results of DCF therapy as induction chemotherapy for borderline resectable T4 cases [6]. DCF therapy was performed in 16 patients, and grade 3–4 leukopenia and neutropenia were observed at a relatively high frequency of 62.5%; however, clinical response was also observed in 10 patients (62.5%). Esophagectomy was performed in 12 of 16 patients, but R0 resection was achieved in 10 (83.3%) patients. The postoperative complication rates of anastomotic insufficiency, pneumonia, and recurrent laryngeal nerve palsy were 16.7%, 25%, and 8.3%, respectively, which were considered acceptable results. They chose esophagectomy under thoracotomy. In the phase II COSMOS study, esophagectomy and reconstruction under an open-chest condition were performed for clinical T4 patients who could be resected by induction DCF therapy. Grade 3–4 leukopenia and neutropenia were found at relatively high frequencies of 41.5% and 66.6%, respectively. As a result, 18 (37.5%) of the 48 cases were judged to be resectable after three courses of DCF therapy. R0 resection was achieved in all cases [24]. In our current study, R0 resection was achieved in 10 (100%) of 10 induction DCF therapy cases, while R0 resection was achieved in 23 (79.3%) of 29 induction FP therapy cases. Although not significant, the usefulness of DCF therapy was presumed to be high, as reported in other institutions. For CS and SS cases, surgery that effectively utilizes the magnified image of a thoracoscope with a small thoracotomy is considered to be useful to achieve R0 resection more reliably. In addition, we performed CS on borderline T4 cases, and resection was performed in tumors that were considered resectable in 29 (59.2%) of 49 patients who received FP therapy and 10 (71.4%) of 14 patients who received DCF therapy. DCF therapy had a higher rate of surgery, but no significant difference was observed (*P* = 0.69). There was no significant difference in survival between the two groups (see Additional file 1). However, on the other hand, in the case of R0 resection, the OS of the FP therapy group was significantly longer than that of the DCF therapy group (see Additional file 2). Further studies are needed on this detail, and it cannot be concluded that FP therapy is superior to DCF therapy in CS.

A limitation of this retrospective study was that it was carried out at a single institution, with a limited number of cases. In the future, to prove the usefulness of this technique, it will be necessary to study this technique jointly with other facilities. In addition, there may be differences between institutions and doctors in determining T4 cases. Therefore, unified diagnostic criteria, such as diagnostic imaging, are necessary to determine whether this surgical method is useful in other facilities.

## Conclusions

We performed esophagectomy with thoracoscopy for CS and SS in our department. The prognosis of patients who underwent surgery with R0 was significantly better, and it was considered important to determine how safe resection surgery can be reliably achieved with R0. The combination of intensive chemotherapy, such as DCF, with surgery may increase the rate of successful R0 resection.

## Supplementary Information

**Additional file 1.** There was no significant difference in survival between the FP group and DCF group.

**Additional file 2.** There was significant difference in survival between the FP group and DCF group in the case of R0 resection.

## Data Availability

The datasets used and/or analyzed during the current study are available from the corresponding author upon reasonable request.

## Abbreviations

SS: Salvage surgery
CS: Conversion surgery
FP: 5-FU and cisplatin
DCF: 5-FU, cisplatin, and docetaxel
CRT: Chemoradiation therapy
CT: Computed tomography
OS: Overall survival
DFS: Disease-free survival
