# Supplementary figures and images for: Efficacy of thoracotomy and thoracoscopic-assisted esophageal surgery in conversion and salvage surgeries: a retrospective study
Source: World J Surg Oncol. 2022 May 23;20:163. doi: 10.1186/s12957-022-02637-8 (PMC9125810; doi:10.1186/s12957-022-02637-8)

## Slide 1
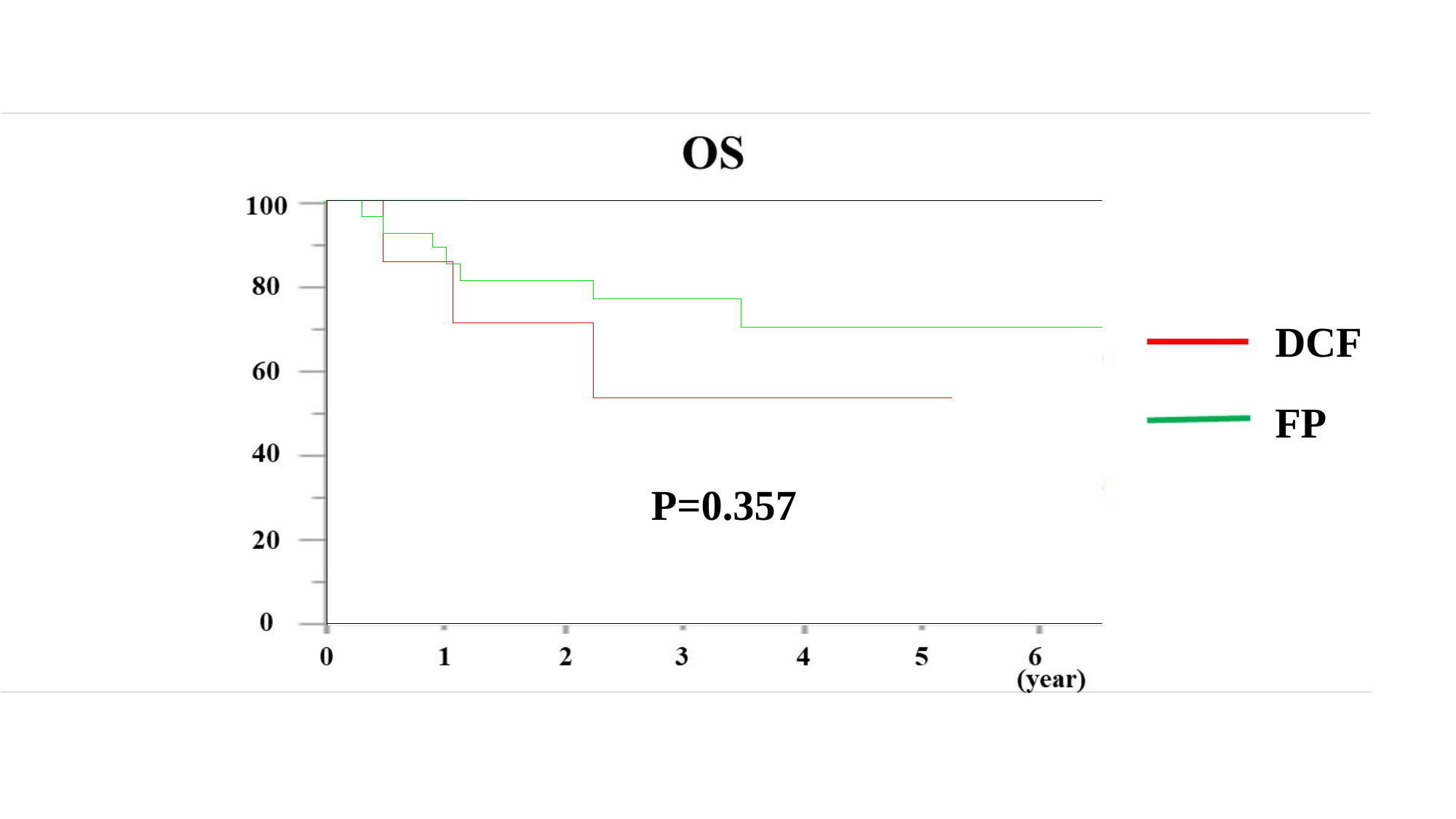

DCF
FP
P=0.357

Supplement: Supplementary file 1 — Additional file 1. There was no significant difference in survival between the FP group and DCF group. [file 12957_2022_2637_MOESM1_ESM.pptx]

## Slide 1
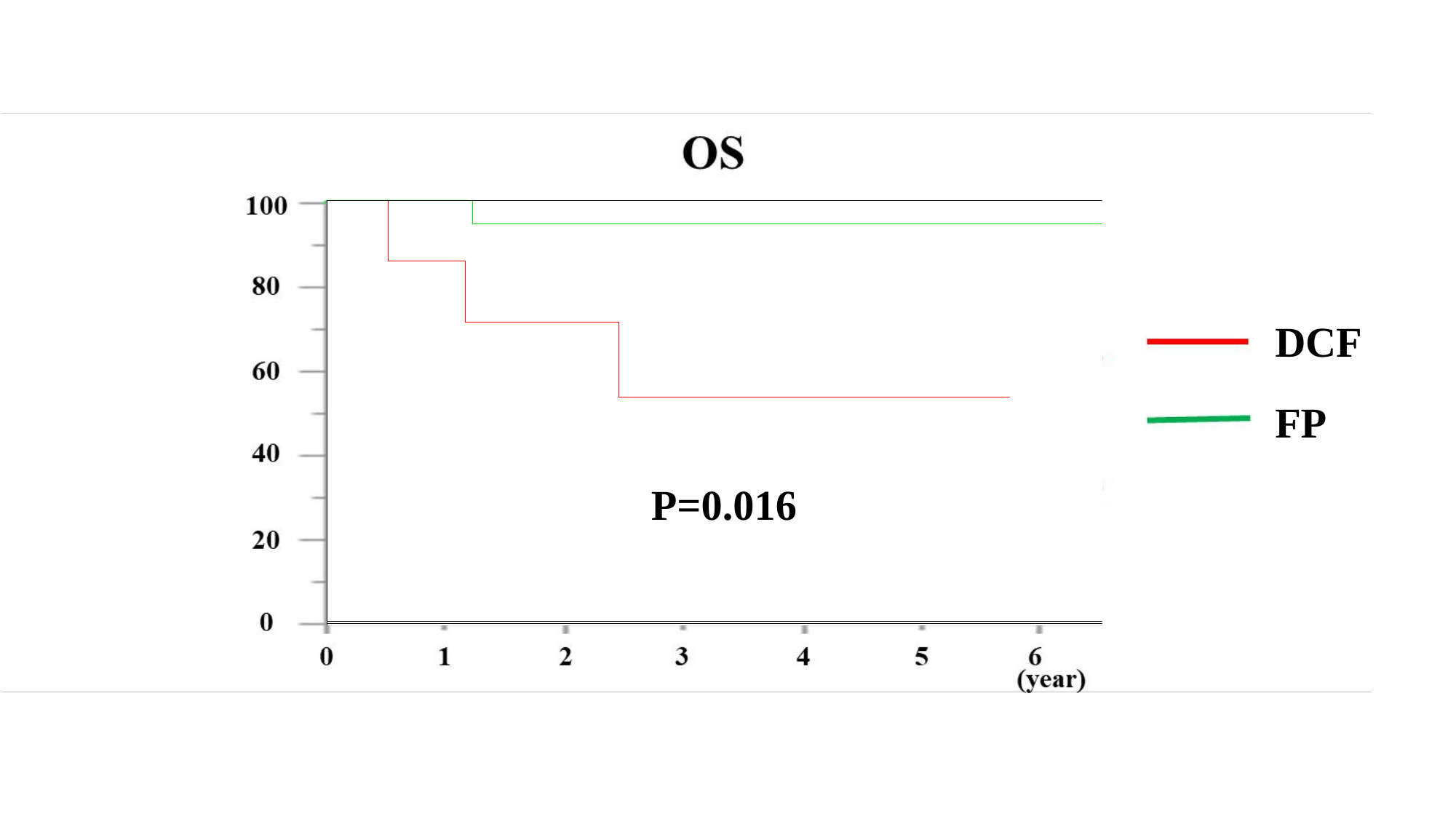

DCF
FP
P=0.016

Supplement: Supplementary file 2 — Additional file 2. There was significant difference in survival between the FP group and DCF group in the case of R0 resection. [file 12957_2022_2637_MOESM2_ESM.pptx]
